# Supplementary material for: Are carbon emissions trading and green financial instruments synergistic? -Comprehensive quantitative research based on content analysis
Source: PLoS One. 2024 Mar 7;19(3):e0298601. doi: 10.1371/journal.pone.0298601 (PMC10919601; doi:10.1371/journal.pone.0298601)
Supplement: S1 Data — (ZIP) [file pone.0298601.s001.zip › Data/地级市Z_F工作报告.zip!/╕╩╦α/╝╬╙°╣╪╩╨/└┤╫╘╣1⁄2╓┌║┼í╛┬φ┐╦╩2╛▌═°í┐_2018.docx]

各位代表：

　　现在，我代表市人民政府，向大会作工作报告，请予审议，并请各位政协委员和列席人员提出意见。

　　一、2017年工作回顾

　　过去一年，面对严峻复杂的经济形势和艰巨繁重的发展任务，在市委的坚强领导和市人大、市政协的监督支持下，我们认真学习贯彻党的十八大、十九大精神，以习近平新时代中国特色社会主义思想为指导，全面落实习近平总书记视察甘肃重要讲话和“八个着力”重要指示精神，按照省第十三次党代会和市第十一次党代会部署，把握稳中求进工作总基调，贯彻新发展理念，积极应对经济下行压力，统筹推进稳增长、调结构、促改革、惠民生、防风险各项工作，较好完成市十届人大二次会议确定的主要目标任务。

　　——经济运行止跌回稳向好。认真落实省委、省政府“三重”“三一”工作部署，针对生产总值、固定资产投资和工业生产增速等主要指标下滑的严峻形势，加强经济运行分析研判和调度，强化市级领导包抓责任制，出台涉企行政事业性“零收费”、项目全程代办、实体经济和非公经济奖励办法，有效扭转了经济下滑局面。预计全年实现地区生产总值206亿元，增长4%；固定资产投资147.5亿元，下降8.1%；规模以上工业增加值93.7亿元，增长4%；一般公共预算收入18.4亿元，增长7.4%；城乡居民人均可支配收入分别达到36558元、17700元，增长9%和7.5%；全市经济呈现止跌回稳向好态势。

　　——工业转型取得重大突破。全力支持酒钢集团改革创新发展，天成彩铝绿色短流程铸轧铝深加工、粉矿悬浮磁化焙烧选矿改造、新型金属复合材料产业化一期等项目基本完成。牙买加165万吨氧化铝项目投料生产。地方工业转型取得实质性突破，一特年产10万辆多功能专用汽车一期建成，完成产值4000余万元。索通预焙阳极二期投产，嘉西光伏产业园110千伏配送工程并网运行，甘肃益工高精度交通用铝板项目有序推进，中核嘉华核技术产业园建设进展顺利。

　　——文旅商体深度融合发展。推进总投资70多亿元的关城景区五大项目，丝博园项目基本完工，旅游联接公路建成通车，世界文化遗产公园（花博园）完成总工程量的三分之二，峪泉古街开工建设。峪泉镇入选全国第二批特色小镇。成功举办第六届国际短片电影展、第二届国际房车博览会、国际铁人三项戈壁挑战赛等系列节会赛事。大力发展商贸流通业，开通大厦、名嘉汇商业综合体、天诚美居文化产业园投运，雄关大厦改扩建、嘉丰源大厦、金翼电商快递物流园等项目进展顺利。全年接待旅游人数857万人次，全社会旅游收入57.2亿元，分别增长22%和26%。

　　——城乡统筹发展深入推进。发挥规划引领作用，城市总体规划获省政府批复实施。打造综合交通枢纽，嘉峪关至黑鹰山战备公路、嘉峪关至祁丰旅游公路相继建成，G312线嘉峪关至清泉段开工建设。完善城市基础设施，建成丝博园景区道路下穿立交、双拥路公铁立交等项目，改造提升文化路、祁连路等老旧道路9条，新建改造给排水、供热、煤气等各类管线76公里。推进城乡融合发展，培育戈壁设施农业，建设日光温室121座。深化农村集体产权制度改革，完成农户土地承包确权6.8万亩、发证率99.2%。改造农村D级危房77户，塔湾村、中沟村获全省“千村美丽”示范村，稳步推进峪泉镇国家级建制镇示范试点建设。

　　——创新驱动发展活力增强。加大科技创新力度，全社会研发经费投入占GDP比重7%，居全省首位。酒钢“2205双相不锈钢研制开发”项目获省级科技进步一等奖，索通“预焙阳极及余热发电”项目获国家优质工程奖。每万人发明专利拥有量从4.39件增加到6.14件。加快科技成果转化，技术市场成交额5.2亿元，增长10%。综合科技进步水平指数达到48%。新增国家知识产权优势企业、高新技术企业9家和省级科技创新型企业、工程实验室、工业设计中心19家。甘肃省名牌产品达到24个。推进大众创业万众创新，省级孵化器和众创空间达到4家，提供“双创”小额贷款担保2.38亿元，增长7倍，创新创业活力逐步增强。

　　——改革开放不断走向纵深。深化“三去一降一补”为重点的供给侧结构性改革，压减生铁、粗钢、水泥落后产能100万吨、140万吨和95万吨，取缔“地条钢”非法企业2户；商品房去库存化率降至10个月；化解政府债务5.7亿元；降低企业成本，减轻纳税人税负4.99亿元，减免23户困难企业税费1000余万元。推进“放管服”改革，加快“互联网+政务服务”建设，实行“一号申请、一窗受理、一网通办”，推行并联审批、联审联批等模式，企业和群众办事更加便捷。深化商事制度改革，新增私营企业1260户、个体工商户3209户，分别增长0.7%和12.6%。不动产登记实现与国家数据汇交，办理不动产权证1.8万余本，有效解决酒钢职工房屋办证遗留问题。成立中小企业金融服务公司，设立城市建设和文化旅游发展两支基金。推行“一企一策”改革清单，国企改革“1+N”制度体系初步形成。围绕“一带一路”举办文化交流活动，依托兰洽会、深圳文博会等平台加大招商引资力度，招商到位资金197.9亿元，增长7.5%。加强对外交流，与约旦佩特拉古城缔结姊妹世界文化遗产地，与北京西城区缔结友好区市。“嘉峪关号”中亚国际货运班列常态化运行。完成进出口总额18.5亿元，增长5.2%。

　　——生态环保力度持续加大。启动“国家森林城市”、“国家生态园林城市”创建工程，完成义务植树9.4万株，退耕还林造林2.68万亩，城乡新增绿地面积451亩，建成区绿地率达到38.3%。狠抓祁连山生态环境问题和中央环保督察反馈问题整改，开展大气、水、土壤污染防治“三大行动”。燃煤锅炉淘汰任务全面完成，餐饮企业炉灶清洁能源改造率98%。酒钢集团实施脱硫、脱硝等节能减排项目，投入近10亿元。全市空气质量优良天数310天、优良率84.9%。市、镇、村三级河长制全面推行，湖长制在全省率先建立。民丰化工土壤污染修复治理项目开工。工业园区循环化改造、国家低碳工业园区试点有序推进。固废综合利用率不断提升。“禁塑”工作有效开展，全社会低碳节约意识正在形成。

　　——民生保障水平全面提升。民生支出占财政总支出的比例达到74.7 %。10项惠民实事基本完成。优先发展教育事业，新建、改扩建校舍面积7.7万平方米、运动场地3.6万平方米，福民、建林街区和南市区3所幼儿园建设进展顺利，中德学院建设全面启动。落实积极就业政策，新增城镇就业8144人，城镇登记失业率2.8%；组织技能培训3607人，输转农村劳动力7175人次；最低工资标准提高10.2%，达到1620元，位居全省前列；农民工工资清欠率93.7%。完善社会保障体系，推行“五险合一”，完成城乡居民医疗保险整合，实现基本医疗保险异地就医直接结算，退休人员平均养老金增长6.3%。补齐医疗卫生短板，南市区医院一期、中医院综合楼主体完工，酒钢医院门诊楼开工建设；重特大病救助由26种扩展到50种，大病报销比例最高达到70%；取消公立医院药品加成，完善分级诊疗制度，有效满足群众就医需求。惠及18个小区、10171户的棚改三年任务全部实施，安置率100%，其中货币化安置34%。严格落实安全生产“党政同责、一岗双责”。扎实推进“国家食品安全示范城市”、“国家农产品质量安全城市”创建。强化社会治安综合治理，推进社会治安立体化防控体系建设，靠实信访维稳责任，矛盾纠纷调处率99.3%，社会大局保持和谐稳定。深化双拥共建，军地军民关系更加融洽。审计统计、物价粮食、防震减灾、人防气象、机构编制、档案市志、外事侨务、老龄妇幼、残疾人事业、社会团体等工作深入开展。

　　——全国文明城市创建成功。举全市之力，决战决胜全国文明城市创建。坚持核心价值观引领，强化宣传教育，建成核心价值观主题广场、主题公园、诚信街、好人街和一批道德长廊。加强城市精细化管理，实现社会治安综合治理、市容卫生、环境保护、安全生产和食品安全网格化管理服务全覆盖。开展全域全城无垃圾“六整治一行动”，陈年老旧垃圾和“牛皮癣”小广告得到有效清理，街面门头牌匾更换整洁，违法违章建筑全面拆除。坚持党政军民学齐上阵，4.6万名志愿者、245支志愿服务团队广泛参与。历经22年不懈努力，我们荣获第五届全国文明城市。这份荣誉承载着30万雄关儿女的追求和夙愿，倾注了全市人民的心血和汗水，成绩和光荣属于每一位钢城人民！

　　过去的一年，我们牢固树立“四个意识”，坚决维护以习近平同志为核心的党中央权威和集中统一领导。全面履行从严治党责任，推进政府系统党风廉政建设和反腐败斗争，坚决肃清王三运流毒和影响。认真落实中央八项规定实施细则精神，扎实开展“三纠三促”专项整治行动，坚持不懈转作风抓落实，狠抓政府自身建设，着力提高政府公信力和执行力。推进依法行政，修订《市政府工作规则》，成立专家决策咨询委员会和信息化建设专家库，制定重大事项向市委报告、重大决策出台前向市人大及其常委会报告制度，主动接受人大法律监督、政协民主监督和社会各界监督。共办理人大代表建议373件、政协委员提案和建议201件，办复率和满意率99%。加强审计监督，出台《审计全覆盖实施方案》，整改落实问题资金10.39亿元。加强行政监察，严肃查处了一批违纪违法案件。加大财力统筹，“三公”经费支出下降7.4%，节约资金全部用于民生领域。

　　各位代表！奋斗充满艰辛，成绩来之不易。这些成绩的取得，是省委、省政府亲切关怀和市委正确领导的结果，是市人大、市政协、嘉峪关军分区、酒钢集团公司、中核四〇四有限公司及驻嘉各单位大力支持的结果，是全市各族人民团结拼搏的结果。在此，我代表市人民政府，向人大代表、政协委员，向广大干部群众，向各民主党派、工商联、无党派人士、人民团体和社会各界人士，向驻嘉部队、武警官兵和驻嘉各单位，表示衷心的感谢，并致以崇高的敬意！

　　各位代表，在总结成绩的同时，我们也清醒地认识到，我市经济社会发展还存在诸多困难和问题。一是经济发展质量不高，投资拉动型和经济结构单一的格局尚未根本改变；二是创新驱动发展能力不足，新旧动能转换较慢，新技术、新模式、新业态发展滞后；三是“放管服”改革和国资国企改革还不到位，“一带一路”向西开放的通道经济和前沿作用发挥不充分；四是城市管理精细化程度不高，巩固全国文明城市成果还有大量工作要做；五是发展不平衡不充分，全面建成小康社会短板突出，非公经济占比不高，城乡融合发展不够；六是一些干部创新不足、能力不足和担当意识不强并存，推拖绕躲问题还不同程度存在。这些问题，我们不回避、不推诿、不懈怠，要主动担当、攻坚克难，采取坚决有力措施加以解决，让人民群众更满意！

　　二、2018年主要任务

　　2018年是贯彻党的十九大精神、落实省第十三次党代会部署的开局之年，是决胜全面建成小康社会、实施“十三五”规划承上启下的关键一年，也是酒钢公司成立60周年，做好今年政府工作意义特殊而重大。在前进的道路上，我们既面临着不少困难与挑战，也面临着前所未有的历史性机遇。党的十九大作出了中国特色社会主义进入新时代和我国社会主要矛盾变化的重要判断，提出优化区域开发布局、加大西部开放力度等重大部署，为我们实现“两个率先”提供了政策支撑；中央和省委经济工作会议强调推动高质量发展，这是确定发展思路、制定经济政策、推动产业发展的根本要求，为我们做好今年经济工作指明了方向；随着“一带一路”战略、对外开放的深入推进，我市交通枢纽地位和物流通道优势更加凸显，为我们加快建设向西开放的桥头堡奠定了坚实基础。只要我们抢抓机遇、顺势而为，就一定能够变被动为主动、化压力为动力，开创经济转型升级、城市转型发展的新局面。

　　政府工作的总体要求是：全面贯彻党的十九大精神，以习近平新时代中国特色社会主义思想为指导，深入贯彻习近平总书记视察甘肃重要讲话和“八个着力”重要指示精神，全面落实省第十三次党代会和市第十一次党代会精神，坚持稳中求进工作总基调，坚持新发展理念，统筹推进“五位一体”总体布局和协调推进“四个全面”战略布局，按照高质量发展要求，以供给侧结构性改革为主线，统筹做好稳增长、促改革、调结构、惠民生、防风险各项工作，促进经济社会持续健康发展，加快实现“两个率先”奋斗目标。

　　经济社会发展主要预期目标是：生产总值增长6.5%左右，固定资产投资增长7.5%，社会消费品零售总额增长8%，一般公共预算收入增长7%，城乡居民人均可支配收入分别增长7%、8%，居民消费价格指数涨幅控制在3%以内，单位生产总值能耗和主要污染物排放完成省级下达的控制指标。上述发展目标，围绕推动高质量发展，把投资有回报、产业有市场、企业有利润、居民有收入、政府有税收、环境有改善作为工作的衡量标准，力争我市经济在质的大幅提升中实现量的持续增长。

　　各位代表，在全省率先全面建成小康社会，是我们对全市人民作出的庄严承诺。一诺千金，时间不等人！我们必须突出问题导向，对标全面小康38项监测指标，抓住薄弱环节，下足“绣花功夫”，加快补齐科技进步贡献率、基层民主参选率、文化及相关产业增加值占GDP比重、公共交通服务指数、工业固体废物综合利用率、森林覆盖率、建成区绿地率、单位GDP能耗的关键短板，确保全面建成小康社会赢得群众认可、经得起历史检验。

　　实现确定目标，我们要重点抓好以下工作。

　　（一）坚持高质量发展，提升项目投资效益。开展“项目建设提质年”活动，发挥项目支撑和引领高质量发展的作用，建好动态基本建设项目、财政项目、招商引资项目和社会资本PPP项目等四个项目库。抓项目谋划，紧扣社会主要矛盾新变化，抓住国家深入推进“一带一路”建设、构建西部大开发新格局等机遇，用足用活国家产业政策，聚焦“三大攻坚战”、民生改善、基础设施、动能转换、财源培植、转型创新等领域，谋划储备一批大项目、好项目。抓项目争取，紧盯国家投资导向和市场环境变化，加强与省级部门的衔接沟通，争取更多资金支持，防范化解政府债务。紧盯重点区域和企业，组织专业骨干人员、依托各类协会商会开展精准招商，积极承接东部产业转移，争取更多项目落地。抓项目落实，强化领导包抓项目责任制，紧扣项目申报、规划选址、用地审批、资金筹措等关键环节，实行“周调度、月通报、季问效、年奖惩”，全力实施嘉北工业园区污水处理厂等57项、总投资458亿元的领导包抓项目，加快南市区初中等56项、当年投资46.9亿元的续建项目，启动S06酒嘉绕城高速等146项、计划投资113.3亿元的新开工项目，努力提高项目开工率、资金到位率、建成投产率。

　　（二）狠抓提质增效，促进工业转型升级。聚焦实体经济发展，深入实施“工业强市”战略，推动质量变革、效率变革、动力变革。

　　改造提升传统产业。全力支持酒钢集团产业转型和产品升级，加快不锈钢铬钢酸洗线、7号高炉优化升级等重点项目建设，开发铬镍系列和食品级、医药级不锈钢等高附加值产品，提升企业核心竞争力。推进铝产业高端绿色发展，加强铝材深加工和轻量化研究，实施甘肃益工25万吨高精度交通用铝板、天成铝箔材加工等项目，打造氧化铝—电解铝—铝精深加工全产业链。推广装配式建筑，争创国家级装配式建筑产业基地和示范城市。发挥一特汽车带动作用，加快汽车产业园建设，引进培育整车零部件生产、改装制造、汽车交易等配套企业，打造汽车产业集群。

　　培育壮大新兴产业。大力发展信息技术、节能环保、清洁能源、先进制造、军民融合等新兴产业，培育新的经济增长极。坚持以信息化带动智能化，实施“互联网+智能制造”行动，推动互联网、大数据、人工智能与实体经济融合发展。加强与中核四〇四、中核龙瑞等企业的战略合作，重点推进核装备设备、核非标设备等研发加工，加大军民融合产业园建设，打造民用核技术产业基地。

　　提升园区发展质量。完善园区企业准入退出机制，依法依规清理“僵尸企业”。完成园区低碳和循环化改造试点任务，加大基础设施配套建设力度，吸引技术含量高、附加值高的企业和项目落户。探索工业用地供应和价格形成新模式，通过“税费控地”、整治“圈而不建”、查处违规用地等措施，盘活闲置土地，提高投资强度和投入产出比，增强园区产业聚集和辐射带动能力。

　　（三）突出富民强市，大力发展第三产业。把文旅商体深度融合作为转型发展的强大引擎，加快发展现代服务业，全力打造“丝绸之路经济带”国际旅游目的地、休闲消费性城市和全国区域级物流节点城市。

　　加快发展旅游产业。按照“景城一体”，统筹抓好全域旅游示范区创建。加快丝绸之路文化博览园、花博园、峪泉古街、游客服务中心、长城博物馆等项目建设，确保世界文化遗产公园如期运营；推进戈壁大峡谷地质公园、草湖国家湿地公园等重点项目和特色小镇建设；开建自驾车旅游服务区、房车露营地，完善旅游标识等配套设施。实行以奖代补鼓励特色旅游商品开发，培育组建集研发、生产、营销于一体的新社会组织。扶持提升乡村民宿和农家乐规模品质，全面启动特色旅游村镇创建工作，打造富有地方特色的乡村旅游示范村，鼓励农民融入旅游创业、围绕旅游增收。

　　推动文化繁荣发展。深度挖掘地域特色文化，实施人文古迹保护和历史再现工程，推进黑山岩画、魏晋文化保护与展示，逐步完善石关峡保护设施，提升民间博物馆群运营水平。加强非物质文化遗产保护。健全文化体育服务设施，改造提升城乡社区体育健身设施和文体活动广场。加快文化创意产业园建设，开发文创产品，发展艺术创造、动漫、多媒体等新业态，构建文化事业和文化产业多元发展格局。

　　强化物流商贸带动。加快推进国际港务区建设，实施酒钢多式联运物流园、铁路综合货场、仓储物流配送中心等项目，提升嘉东五大市场投入产出效益。培育现代物流企业，发展第三方物流，壮大物流经济和通道经济。加快紫轩国际、雄关大厦等城市综合体和商贸中心建设，促进商贸服务业提档升级。推动中核龙瑞、中核地质研究院办公研发和生活基地落地，增强城市活力。

　　（四）推动乡村振兴，加速城乡融合发展。按照“产业兴旺、生态宜居、乡风文明、治理有效、生活富裕”的要求，实施乡村振兴战略，提高新型城镇化发展水平，推动农业强、农民富、农村美。

　　推进农村“三变”改革。落实农村土地集体所有权、农户承包权、土地经营权“三权分置”。开展农村资源变资产、资金变股金、农民变股东改革。加快农村集体、农户清产核资和确权工作，整合各类财政专项资金和其他涉农资金，制定具体折股量化办法，建立股权收益分配和分红机制，让农民获得更多财产性收入，不断壮大村集体经济。鼓励农民以股权与专业大户、专业合作社、农业龙头企业等新型经营主体结成紧密的利益共同体，使农村闲置的资源活起来、分散的资金聚起来、群众的日子好起来。

　　培育特色优势产业。发展精品高效农业，加快实施文殊镇戈壁现代农业示范园、新城镇田园综合体、峪泉镇高效生态种植园等项目。推进农村一二三产深度融合，引导农户发展交通运输、餐饮住宿、电子商务等产业，为持续稳定增收提供支撑。加强现代农业科技推广，继续实施高标准农田改造和土地整理，增加农业科技含量和附加值。加大病虫害治理，扩大绿色有机净菜加工配送规模，推动国家农产品质量安全市建设。

　　加快生态文明小康村建设。开展农村人居环境整治三年行动，实施三镇污水处理、农村垃圾收集处理、改厨改厕、后院整治等项目，不断改善村容村貌和人居环境。巩固提升饮水安全、农村公路、乡村造林、居住条件，深化美丽宜居乡村建设。做实农村网格化社会治理，培育塑造文明乡风，健全自治、法治、德治相结合的乡村治理体系，让农村成为安居乐业的美丽家园。

　　（五）聚力创新发展，加快动能接续转换。围绕促转型、提质量、增效益，实施创新驱动发展战略，不断激发全社会创新活力。

　　增强创新创业能力。加大政府资金投入，引导企业增加研发投入，弘扬企业家精神和工匠精神，构建以企业为主体的技术创新体系，创建更多国家和省级科技创新中心、重点实验室、技术研发中心。在新能源、新材料、高端装备制造、信息技术等领域，支持企业联合高校院所组建柔性产学研用联盟，培育更多“科技小巨人”企业。搭建河西走廊区域性科技成果交易服务平台，促进重大科研成果转化，提高科技进步贡献率。加快建设创新创业平台和孵化基地，建成一批低成本、便利化、开放式的众创空间，全面提升“双创”服务能力。

　　强化创新人才支撑。完善引进人才职称待遇、住房保障、子女就学、经费支持等方面优待政策，实行“一人一策”，提高人才引进精准性。继续实施领军人才、高层次人才、创新创业青年人才等重大工程，聚焦新材料研究、金融信息、城市规划、医疗教育等领域引进培养专业技术、经营管理等实用型人才。坚持不求所有、但求所用的柔性引才方式，深化与高等院校、科研机构、国家级智库的战略合作，发挥专家咨询委员会作用，强化人才对经济社会发展的智力支撑。

　　（六）深化改革开放，激发内生动力活力。加大重点领域和关键环节改革力度，深度融入“一带一路”建设，为经济社会发展增活力添动力。

　　深化“放管服”改革。加大简政放权力度，在继续取消一批行政审批事项的基础上，实行“一窗办、一网办、简化办、马上办”，成立行政审批局，最大限度缩短审批时限。以信息化手段推进“互联网+政务服务”，开展企业全程电子化登记，推动利企便民的服务事项网上办理，真正让数据多跑路、群众少跑腿。坚持“先照后证”“多证合一”，强化事中事后监管，实现“双随机、一公开”监管全覆盖。落实降低企业成本“40条”措施，实行涉企行政事业性“零收费”试点。发挥地企联席会议作用，建立与酒钢集团、中核四〇四、中核龙瑞等企业重大问题会商机制。深入开展“营商环境建设年”活动，加快构建新型“亲”“清”政商关系，主动当好“店小二”“星级服务员”，帮助企业爬坡过坎。坚决对破坏营商环境行为“零容忍”、严惩处。

　　推进国资国企改革。以公司制改革、脱钩改制、整合重组、发展混合所有制经济为重点，“一企一策”分类推进国资国企改革。完善现代企业制度，加快建立法人治理结构，增强企业活力和市场竞争力。促进国有企业股权减持、股权多元化，加快放大国有资本功能。推动国资监管机构全面履职，加强市属国有企业资产集中统一监管，确保国有资产保值增值。加快在嘉企业“三供一业”和其他办社会职能分离移交，推动国有企业瘦身健体、固本培元。

　　加大对外开放力度。抢抓“一带一路”战略机遇，做实做细开放枢纽、通道经济、产能合作、文化交流的文章，真正成为甘肃向西开放的龙头和前沿。建成霍尔果斯联络处，打开向中西亚开放门户，积极融入中新南向通道建设。支持酒钢集团参与境外资源合作开发，扩大特色优势产品出口份额。推进嘉峪关机场改扩建，争取设立航空口岸和海关特殊监管区。办好第八届“敦煌行·丝绸之路国际旅游节”开幕式、第七届国际短片电影展、第三届国际房车博览会等节会赛事，提升对外开放的广度、深度和城市知名度。

　　（七）聚焦精细管理，完善城市服务功能。统筹城市规划、建设和管理，加快城市发展由外延扩张向内涵提升转变，打造精致、特色、活力、文明的现代化城市。

　　强化规划引领作用。加快完善“多规合一”体系，推动城市总体规划和土地利用总体规划“两图合一”，抓紧编制和修订各类专项规划，构建城市“成长坐标”体系。科学划定“三区四线”，合理确定城市规模、开发边界、开发强度和保护性空间，坚持一张蓝图绘到底。强化城市设计，在主要街区构置体现丝路风情和地域文化的特色亭台、水域景观、经典雕塑，促进城市与环境、人文与自然和谐共生。

　　完善城市服务功能。统筹新区开发建设和老旧城区提升改造，完善中心城区功能，提高城市空间和土地利用效率。打造立体交通体系，推进嘉峪关至祁丰战备公路、G312线嘉峪关至清泉段、S06酒嘉绕城高速建设，做好S215线嘉峪关至二指哈拉公路提级改造项目前期工作。加快电力通信、给水排水、燃气供热、污水处理等基础设施建设，推进南市区道路、金港路清嘉高速桥梁、讨赖河人行景观桥等项目，实施五一路、建设路、雄关路等老旧道路和镜铁市场、同乐市场提升改造工程。完善覆盖城乡的交通运输网络，提升公共交通服务指数。

　　加强城市精细管理。推进城市综合管理立法，提交市人大力争年内出台《市容和环境卫生管理条例》。优化市政公用、市容环卫、园林绿化等行政职能，统筹市政设施集中高效运营。整合全市信息化资源，建成大数据中心，推进新型智慧城市建设。制定巩固全国文明城市创建三年行动计划，开展全域全城无垃圾三年专项整治行动，加强城乡结合部、背街小巷等重点区域整治。全面推进“厕所革命”，统筹城乡和旅游景区厕所建设。坚持共建共享，激发市民主人翁意识，让随手文明、爱城护城成为广大市民的自觉行动，形成“人民城市人民建”的浓厚氛围。

　　（八）推进绿色发展，持续建设美丽家园。牢固树立“绿水青山就是金山银山”理念，坚决守住生态功能保障基线、环境质量安全底线、自然资源利用上线，让嘉峪关的天更蓝、地更绿、水更清、城更美。

　　加大城乡绿化力度。开展城区提质扩绿行动，坚持拆墙透绿、见缝插绿、层次分明、疏密有度，提升公共空间园林绿化质量，推动绿化达标小区、花园式单位创建，重点实施五一路、迎宾路等13条道路绿化提升改造，建成区绿地率提高到38.9%以上。开展乡村绿化行动，推进农村“四旁”绿化、乡村道路绿化、宜林地工程造林，逐步提升农村生态环境质量。开展大规模国土绿化行动，在城乡结合部、公路沿线、工业园区等重点区域开展义务植树，鼓励企业建设园林式厂区，提高城乡森林覆盖率。

　　强化生态环境保护。落实省级环保督察整改任务，深度推进污染防治。实施草湖湿地生态修复、讨赖河水土保持、城乡周边防风林带和防风固沙等项目，共同构筑祁连山生态安全屏障。深入推进大气、水、土壤污染防治“三大行动”。严控污染物总量排放，推进燃煤锅炉、扬尘污染、餐饮油烟、噪音污染整治。全面落实河长制、湖长制责任。建成嘉北工业园区污水处理厂。强化生态环境执法，始终保持严厉打击环境违法行为的高压态势。

　　推进低碳循环发展。抓好工业、建筑、交通、公共机构等领域节能减排，降低能耗强度，提高能源利用效率。支持酒钢润源公司“危废”处理，推进固体废弃物回收利用、生活垃圾分类回收和餐厨垃圾无害化处理，加快再生资源利用产业化。聚焦构建生态产业体系，加强项目节能评估和环境影响评价，引进低能耗、高效率、污染小的科技型项目，有效防范环境污染风险。增强全民环保意识，倡导绿色低碳发展方式和生活方式，构建政府主导、企业主体、社会组织和公众参与的环境治理体系。

　　（九）持续改善民生，不断增进民生福祉。坚持以人民为中心的发展思想，把老百姓的安危冷暖时刻放在心上，不断满足人民日益增长的美好生活需要。

　　坚持教育事业优先发展。扩大公办和普惠性民办幼儿园覆盖面，建成福民街、建林街幼儿园和南市区幼儿园，着力解决学前教育优质资源不足的问题。推进义务教育优质均衡发展，实现“全面改薄”圆满收官，建成南市区小学和胜利路小学、师范附属学校综合教学楼，开工建设南市区初中、实验中学改扩建等项目，逐步解决城区中小学“大班额”问题。整合职业教育资源，加快中德学院建设步伐。

　　提高就业质量和收入水平。持续开展职业技能培训，促进创业带动就业，全年新增就业8000人左右，城镇登记失业率控制在3%以内。做好高校毕业生、农村转移劳动力、下岗失业人员、退役军人等群体就业，确保零就业家庭动态“清零”。落实农民工工资支付保障制度，下功夫解决工资拖欠难题。逐步解决同工不同酬问题。提高居民劳动收入，拓宽财产性收入渠道，让群众的收入有增长、生活有改善。

　　持续完善社会保障体系。强化以“养老、医疗、失业、工伤、生育”五项保险为重点的基本保障。加快推进社会保险信息化建设，使城乡居民办事更加快捷方便。完善城乡低保制度，更加关注残疾人、老年人、留守儿童等困难弱势群体。为低保范围内的重度残疾人家庭发放生活补贴。为60岁以上老年人购买意外伤害保险。启动新三年棚改计划，年内实施危房拆迁1623户、危房改造12189户。加快建立多主体供给、多渠道保障、租购并举的住房制度，满足各类群体住房需求。

　　推进健康嘉峪关建设。深化公立医院综合改革，推进医疗、医保、医药“三医”联动改革，健全医师多点执业、紧密型医联体、分级诊疗等机制。加快实施南市区医院、中医院二期、酒钢医院门诊楼等项目，提升医疗卫生服务能力。全力创建国家食品安全示范城市，建成“三检”中心、生猪定点屠宰厂，实现食品从农田到餐桌、药品从生产到使用全过程监管。加快建设多层次、智能化、有标准、有质量的医养服务体系，努力提高城乡社区养老服务水平。

　　维护社会和谐稳定。深入推进依法治市，实施“七五”普法规划，增强全民法治意识。深化社会治安综合治理立体化防控体系建设，建成公安情报合成作战中心，提升社会治安管控能力。依法打击金融违法违规活动。加强诚信体系建设，联合惩治失信行为。强化信访维稳和矛盾纠纷排查化解，有效解决群众合理诉求。推进安全生产领域改革和城市安全发展，加大隐患排查治理力度，防范和遏制重特大事故发生，保障人民群众生命财产安全。加强城乡社区治理，增强居民自治能力，提升基层民主参选率。推动军民深度融合发展，争创双拥模范城全国“七连冠”和全省“九连冠”。

　　继续办好惠民实事。从解决群众普遍关心的突出问题入手，想群众之所想，急群众之所急，办好高铁南站电梯配置、推进城乡公厕建设、完善全民健身设施、重点公共区域免费WIFI全覆盖、“互联网+政务服务惠民”、推行雄关便民卡、三镇通公交、关城景区向市民常年免费开放8件惠民实事，持续提升群众获得感和幸福感。

　　三、全面加强政府自身建设

　　新时代、新征程、新使命，对政府工作提出了新的更高要求。我们必须把政府自身建设摆在更加突出的位置，坚持以政治建设为统领，全面提升政府履职能力，努力建设讲政治、敢担当、有作为、守清廉的人民满意政府。

　　提高政治站位，建设学习政府。认真学习贯彻党的十九大精神和习近平新时代中国特色社会主义思想，扎实推进“两学一做”学习教育常态化制度化，深入开展“不忘初心、牢记使命”主题教育，引导党员干部牢固树立“四个意识”，严格遵守政治纪律和政治规矩，坚决同以习近平同志为核心的党中央保持高度一致，把对党忠诚、为党尽职、为民造福作为根本政治担当。坚持学思践悟、知行合一，广泛学习履行岗位职责必备的业务知识，转化为发展思路和工作方法，更好担负起推动经济社会发展的重任。

　　坚持解放思想，建设效能政府。把解放思想作为破解难题、推动发展的总开关，在政府系统开展“解放思想大讨论”活动，摆脱思维定势、传统观念和固有经验束缚，勇于变革、勇于创新，永不僵化、永不停滞。开展“能力素质大提升”活动，围绕增强“八个本领”，注重培养专业能力、专业精神，有针对性地举办项目谋划推进、审计监督、统计监测等领域专项培训，弥补知识短缺和能力短板。开展“行政效能大提速”活动，少讲不能办、多想如何干，坚决纠正办事拖沓敷衍、责任上推下卸、工作畏首畏尾等懒政怠政行为，提升政府执行力和公信力。

　　推进依法行政，建设法治政府。坚持把法治思维和法治方式贯穿政府工作全过程，依法履行职责，做到法定职责必须为、法无授权不可为。落实公职律师、法律顾问、专家咨询等制度，严格执行重大行政决策法定程序，推动政府依法、科学、民主决策。主动接受人大法律监督、政协民主监督、司法监督和社会舆论监督，认真办理人大代表建议和政协委员提案。继续加大政务公开和信息公开力度，完善新闻发布制度，提高工作透明度。

　　持续转变作风，建设服务政府。严格落实中央八项规定实施细则精神，坚决整治“四风”突出问题和损害群众利益的不正之风。始终把人民群众对美好生活的向往作为奋斗目标，畅通民意表达渠道，及时回应社会关切，真心实意为群众办实事、解难事。坚持实事求是，大兴调查研究之风，做到各项工作情况明、底数清，确保决策精准、措施有效。强化责任担当，不摆花架子，不搞形式主义，沉下心来、扑下身子，以钉钉子精神把各项工作落实落细落小。

　　落实主体责任，建设清廉政府。落实十九届中纪委二次全会精神，认真履行管党治党主体责任，全面落实“一岗双责”，加强政府系统党风廉政建设。教育党员干部强化廉洁自律意识，自觉清洁“工作圈”“社交圈”“亲友圈”，时刻保持思想纯洁、行为廉洁，筑牢拒腐防变堤坝，守住为官从政底线。规范行政权力运行，在公共资金使用、国有资产运营、工程招标投标、土地出让、行政审批等重点领域，建立严格的监督制约机制，让权力在阳光下运行，努力实现干部清正、政府清廉、政治清明。

　　各位代表！转型发展重任在肩，全面小康催人奋进。让我们更加紧密地团结在以习近平同志为核心的党中央周围，在省委、省政府和市委的坚强领导下，在市人大、市政协的监督支持下，紧紧依靠全市人民，不忘初心、牢记使命，攻坚克难、砥砺前行，为推动我市在全省率先全面建成小康社会，率先实现转型跨越发展而努力奋斗！
